# Risk Factors for Anaplastic Thyroid Cancer

**DOI:** 10.1155/2014/815070

**Published:** 2014-05-18

**Authors:** V. Zivaljevic, N. Slijepcevic, I. Paunovic, A. Diklic, N. Kalezic, J. Marinkovic, R. Zivic, B. Vekic, S. Sipetic

**Affiliations:** ^1^Centre for Endocrine Surgery, Clinical Centre of Serbia, Koste Todorovica 8, 11000 Belgrade, Serbia; ^2^School of Medicine, University of Belgrade, Dr Subotica 8, 11000 Belgrade, Serbia; ^3^Institute of Medical Statistics and Informatics, School of Medicine, University of Belgrade, Dr Subotica 8, 11000 Belgrade, Serbia; ^4^Clinical Centre “Dr Dragisa Misovic”, Heroja Milana Tepica 1, 11000 Belgrade, Serbia; ^5^Institute of Epidemiology, School of Medicine, University of Belgrade, Visegradska 26A, 11000 Belgrade, Serbia

## Abstract

*Background.* Anaplastic thyroid cancer (ATC) is a form of thyroid cancer with very poor prognosis, but is fortunately quite rare. Its aetiology is unknown and not well researched. *Aim.* The aim of this study was to identify potential risk factors for ATC. *Material and Method.* Case-control study of 126 ATC patients (77 females and 49 males) and 252 controls individually matched by gender, age, and place of abode. In statistical analysis we used a Cox regression model. *Results.* Univariate logistic regression showed that the risk factors for ATC are low education level, type B blood group, goitre, other nonthyroid malignancies, diabetes, late menarche, and an early first pregnancy. Multivariate logistic regression analysis showed that independent risk factors for ATC are low education level (OR = 1.42, 95% CI = 1.09–1.86), type B blood group (OR = 2.41, 95% CI = 1.03–5.66), and goitre (OR = 25–33, 95% CI = 5.66–126.65). *Conclusion.* Independent risk factors for ATC are: low education level, type B blood group, and goitre.

## 1. Introduction

Anaplastic thyroid cancer (ATC) is one of the most aggressive tumours known in medicine. Even though a multimodal approach is used in treating these patients, average survival rate is expressed in months, and one-year survival is 15% [1, 2]. Fortunately, ATC is a very rare malignancy with an incidence of roughly 2 per 1,000,000 residents. Although ATC represents only 2% of all thyroid cancers, it is the cause of death in half of patients whose definitive cause of death is thyroid malignancy [3]. Given that ATC is a very rare malignancy, it has not been extensively researched. Risk factors for ATC have not been sufficiently studied, and its aetiology essentially remains unknown. There have been numerous case-control studies for all histopathological types of thyroid cancer, even medullary thyroid cancer [4, 5]. Apart from our previous study, we did not encounter in the literature other case-control studies of ATC; even though being such an aggressive tumour, it attracts great attention in oncology and endocrinology. Probably, one of the possible reasons for this is that a long period of observation is required to gather a significant number of cases, given that ATC is quite rare. We published the first part of our research, with a smaller number of cases and one control group, ten years ago [6]. Following that study, this research was continued and we published our results with a greater number of cases and two control groups. The first control group consisted of patients with papillary thyroid cancer, while the second control group was formed from patients with goitre [7, 8]. In this paper we will briefly recapitulate the results of our previous studies and present our new results where the control group was formed from examinees from the general population. This paper gives the final results of our long-time research that aimed to identify potential risk factors for the development of ATC.

## 2. Material and Method

A case-control study was used to identify potential risk factors for ATC. The study group consisted of 126 consecutive newly discovered patients with ATC. The diagnosis of ATC was based on the definite histopathological findings for operated patients (55) and on the basis of the results of fine needle aspiration biopsy for patients that were not operated on (71). All patients were diagnosed at the clinic where the study was carried out, from 1993 to 2005. The control group was formed from the general population with the same place of residence as the patients from the studied group. There were twice as many examinees in the control group, which amounts to 252. Examinees that were included in the control group were chosen from neighbours of patients from the studied group in the exact same manner. They were individually matched by place of abode, gender, and age (±2 years). All examinees (cases and controls) were interviewed by the same doctor. Before the interview, all examinees were made familiar with the aim of the study and voluntarily decided whether they would take part in the research. Nobody refused to participate in the study. A specialized epidemiological questionnaire was used. It consisted of questions regarding sociodemographic characteristics, habits, life in endemic goitre areas, exposure to radiation, and professional exposure to chemicals. The next batch of questions consisted of questions about personal history in regard to previous thyroid diseases and thyroid function, other endocrine disorders, other malignancies, various health disorders, diagnostic and therapeutic procedures, previous operations, and use of medicaments. Another group of questions examined family history in regard to thyroid diseases, other diseases of endocrine glands, malignant tumours, and hereditary diseases. Also, detailed data was gathered for menstrual, hormonal, and reproductive characteristics of female examinees. If a significant health disorder was reported, it was double checked in available medical documentation; or, if not available, it was double checked with the closest siblings. In statistical analysis we first used conditional univariate logistic regression analysis (ULR). All variables that were statistically related to ATC, at a level of significance of *P* ≤ 0.10, were included in a multivariate conditional logistic regression analysis model (MLR), to identify independent risk factors for ATC.

## 3. Results

In Table 1 basic sociodemographic characteristics, blood type groups, and habits of examinees are shown. Most of the cases were female with a ratio of 1.5 : 1. In the cases group, age ranged from 37 to 88 years, with an average age of 67 years. Only 10% of cases were younger than 50 years, and only one case was younger than 40 years. More than half of the cases were in their seventh decade (51.2%). More patients resided in an urban area (69.8%) than in a rural area (30.2%). Most cases and even controls had completed only primary school (52.4% and 44.0%, resp.) or had not completed primary school (30.2% and 24.6%). In general, cases had a lower education level (only partial or full primary school) compared to the controls, and this was statistically significant according to ULR. A statistical significant difference was observed in the number of examinees with blood group type B. This blood group was present in 10.3% of cases and 4.4% of controls. Of the 13 cases with type B blood group, twelve had B+, while one had B− blood group. We did not find any statistically significant associations within the habits of the examinees (cigarette smoking, alcohol, and coffee consumption).

Personal and family history of examinees is presented in Table 2. Life in an endemic goitre area did not prove to be statistically significant. Among the cases, 30.2% had a previously diagnosed goitre or thyroid nodules, on average for 18 years (ranging from 1 to 50 years). Six cases had a previous thyroid operation (three for well-differentiated thyroid cancer and three for benign thyroid diseases). On average, the time between the operation and ATC presentation was a bit longer than five years. There was not a single examinee with goitre in the control group, except for one with hyperthyroidism and one with Hashimoto thyroiditis. One of the cases, who had been previously operated for well-differentiated thyroid cancer, received I131 therapy, before the anaplastic transformation occurred. Five patients in the cases group were on substitution or suppressive therapy with L-thyroxine. We found that examinees from the cases group had twice as many nonthyroid malignancies (8.7% versus 4.4%) than the examinees from the control group. This proved to be statistically significant, according to ULR. The most common malignancy was cervical cancer (three cases) and skin cancer (basocellular cancer) in three cases. This was followed by breast cancer (two cases), lung cancer (one case), colorectal cancer (one case), and prostatic cancer (one case). Professional exposure to chemicals was not statistically significant. In comparison to the control group, there were twice as many examinees in the cases group with diabetes, and according to ULR this difference was statistically significant. Even though cases were exposed to radiotherapy more often than controls (1.6% versus 0.4% as it is in Table 2), according to ULR this difference was not statistically significant. A family history of thyroid diseases or other malignancies did not show statistical significance. Hormonal and reproductive characteristics of female examinees are presented in Table 3. Menarche was more common in both of the groups (cases and controls), before the age of 15 years. The number of female examinees who had menarche at the age of 15 years or later was much greater in the cases group (20.8% versus 9.1%). A statistical difference was not observed, according to ULR, in relation to normal menstruations, pregnancy, number of children, number of miscarriages, and deliberate terminations of pregnancy. The same applied to the use of oral contraceptives and menopause estrogens. In the cases group, 19.2% of examinees were pregnant before the age of 19, while in the control group there were 9.2%, and this proved to be a statistically significant difference. Variables that were, according to ULR, statistically significantly related to ATC were included in a MLR model. These are education level, type B blood group, goitre, personal history of nonthyroid malignancies, diabetes, late menarche, and an early first pregnancy. The results of MLR are shown in Table 4. According to MLR three variables are independently related to ATC and represent independent risk factors for ATC: low education level, type B blood group, and goitre.

## 4. Discussion

According to the results of this study, when the control group was formed from the general population, independent risk factors for ATC were low education level (OR = 1.42, 95% CI = 1.09–1.86), type B blood group (OR = 2.41, 95% CI = 1.03–5.66), and goitre or thyroid nodules (OR = 25.33, 95% CI = 5.66–126.65). A low education level is probably a risk factor for ATC for two reasons. People with a lower level of education have generally lower health awareness, and they rarely and/or much later seek medical attention. Also, adequate health care is less frequently available. Bakiri et al. and Gaitan et al. point out that a lower education and a lower socioeconomic status are associated with a higher incidence of ATC and that other thyroid cancers are usually discovered in a more advanced stage [9, 10]. The importance of education is also observed in the paper of Memon et al., where they find that twelve or more years of education significantly decreases the risk of thyroid cancer (OR = 0.6, 95% CI = 0.3–0.9) [11].

Searching the available literature, we did not encounter any papers that associate type of blood group with ATC or any other histopathological type of thyroid cancer. On the other hand, a link between type of blood group and other malignancies has been described. Su et al. find that type B blood group increases the risk of oesophageal cancer, especially in men [12]. Type A blood group is associated with a risk for cancer for various localisations, such as gastric cancer (especially in patients with a positive family history), cancer of the larynx and hypopharynx, pancreatic cancer, breast cancer, and rectal cancer [13–18]. According to the study of Marinaccio et al., type A blood group, apart from increasing the risk for cervical and ovarian cancer, is a bad prognostic factor for these types of cancer [19].

We will now compare the results of this study with the results of our previous studies that also researched risk factors for ATC, but with different control groups. Our first case-control study of risk factors for ATC had a slightly smaller number of cases (110) and one hospital control group [6]. According to that study, independent risk factors for ATC were goitre (OR = 37.55, 95% CI = 4.86–290.11), life in an endemic goitre area (OR = 2.56, 95% CI = 1.05–6.22), a personal history of other nonthyroid malignancies (OR = 5.51, 95% CI = 1.04–29.25), diabetes (OR = 4.06, 95% CI = 1.29–12.81), and a lower level of education (OR = 2.44, 95% CI = 1.17–5.06). Our present study identified three independent risk factors for ATC, compared to our previous study where we identified five. Two of these risk factors are the same in both studies, and those are a lower level of education and goitre. Our present study could not identify life in an endemic area as a risk factor, since the control group was matched by place of abode. Practically, the main differences between these two studies are that one found diabetes and a personal history of other nonthyroid malignancies as independent risk factors, while the other identified type B blood group as an independent risk factor for ATC. Similar results were obtained when we conducted a case-control study with the same 126 cases of ATC that were included in the present study, but the control group was formed from 252 patients with goitre [8]. In fact, the development of ATC is preceded by long-term goitre in nearly half of patients [20]. This is one of the main reasons that we decided to form the control group from patients operated for goitre whose definite pathohistological findings confirmed a benign disease. This allowed us to establish potential risk factors for the development of ATC in patients with goitre. These risk factors could further help us, as additional criteria, when deciding which patients with benign goitre should undergo operative treatment. When patients operated for goitre were used as the control group, there were five variables independently related to the development of ATC. Among these were a lower level of education (OR = 1.85, 95% CI = 1.21–2.82) and type B blood group (OR = 3.69, 95% CI = 1.10–12.49), which were identified in our present study as well, using a different control group. Apart from these two factors, the other three factors were a personal history of other nonthyroid malignancies (OR = 4.37, 95% CI = 1.11–17.31), late menarche, at the age of 15 or later (OR = 2.63. 95% CI = 1.15–5.88), and an early pregnancy, before the age of 19 (OR = 2.96, 95% CI = 1.26–6.96). These two reproductive factors, as found in our present study, were also statistically significantly associated with ATC, according to ULR, but were not found to be independent risk factors. A late menarche and an early pregnancy have been identified as a risk factor for thyroid cancer in women, where its incidence is several times higher than in men [21–23].

Significantly different results were found when the control group consisted of the same number of cases with ATC (126) but where the controls were formed from examinees with papillary thyroid cancer [7]. ATC can develop from preexistent well-differentiated thyroid cancer, which is found in 18–71% of cases with ATC [24–26]. Since, in a significant number of cases with ATC, well-differentiated thyroid cancer has been proven to coexist, on definitive histopathological findings, we decided to form the control group from patients with papillary thyroid cancer, to try and identify which factors could be responsible for the dedifferentiation, that is, anaplastic transformation of well-differentiated thyroid cancer. Based on the results of MLR, when the control group consisted of patients with papillary thyroid cancer, age proved to be the only independent risk factor for the development of ATC (OR = 1.11, 95% CI = 1.05–1.15). Therefore, in comparison to other observed variables, there were no statistically significant differences between patients with ATC and patients with papillary thyroid cancer.

At the end, we would like to point out that in our case-control research of risk factors for ATC we tried to surpass one of the greatest problems in the design of case-control studies and that is the choice of the control group. We overcame this problem by forming several control groups with different criteria and with a bigger number of controls than cases. However, one of the greatest problems of these studies is the fact that they consist of a relatively small number of cases with ATC; but, on the other hand, there are just a few centres in the world that published papers with more than 100 cases of ATC, and unfortunately they have not conducted any case-control studies for risk factors for ATC. Therefore, the results of our study should be tested in a study with a higher number of cases, which could only be achieved by a multicentric study with several high volume centres in the world.

## Figures and Tables

**Table 1:** Sociodemographic characteristics, blood group type, and habits of examinees.

	Cases		Controls		P*
	*N*	%	*N*	%	
Total	**126**	**100.0**	**252**	**100.0**	
Gender					
Males	49	38.9	98	38.9	Matched
Females	77	61.1	154	61.1	
Age					
<40	1	0.8	2	0.8	Matched
41–50	10	8.0	16	6.4	
51–60	30	24.0	40	16.0	
61–70	65	51.2	150	60.0	
71–80	19	15.2	42	16.0	
80+	1	0.8	2	0.8	
Place of abode					
Rural	38	30.2	76	30.2	Matched
Urban	88	69.8	176	69.8	
Education					
No education	38	30.2	62	24.6	0.0124
Primary school	66	52.4	111	44.0	
Secondary school	17	13.5	59	23.4	
High education	5	4.0	20	7.9	
Occupation					
Housewife	38	30.2	80	31.7	0.3948
Farmer	18	14.3	19	7.5	
Blue collar worker	12	9.5	17	6.7	
White collar worker	8	6.3	31	12.3	
Pensioner	48	38.1	93	36.9	
Professional	2	1.6	12	4.8	
Marital status					
Unmarried	4	3.2	2	0.8	0.2222
Married	99	78.6	213	84.5	
Divorced	0	0	7	2.8	
Widow	23	18.3	30	12.0	
Blood group type					
O+	52	41.3	114	45.2	0.0066
A+	54	42.9	120	47.6	
B+	14	10.3	11	4.4	
AB+	7	5.6	7	2.8	
Smoking status					
Smoker	43	34.1	98	38.9	0.3671
Nonsmoker	83	65.9	154	61.1	
Former smoker					
Yes	5	7.1	15	9.7	0.5111
No	78	92.9	139	90.3	
Alcohol					
Yes	3	2.4	2	0.8	0.2254
No	123	97.6	250	99.2	
Coffee					
Yes	110	87.3	222	88.1	0.8240
No	16	12.7	30	11.9	

*Univariate logistic regression analysis.

**Table 2:** Personal and family history of examinees.

	Cases		Controls		P*
	*N*	%	*N*	%	
Total	**126**	**100.0**	**252**	**100.0**	
Life in endemic goitre area					
Yes	29	23.0	47	18.7	0.3190
No	97	77.0	205	81.3	
Goitre					
Yes	38	30.2	0	0	0.0000
No	88	69.8	252	100.0	
Hyperthyroidism					
Yes	1	0.8	0	0	0.6623
No	125	99.2	252	100.0	
Thyroiditis					
Yes	1	0.8	0	0	0.6623
No	125	99.2	252	100.0	
I131 therapy					
Yes	1	0.8	0	0	0.6623
No	125	99.2	252	100.0	
L-Thyroxin therapy					
Yes	5	4.0	0	0	0.1858
No	121	96.0	252	100.0	
Nonthyroid malignancies					
Yes	11	8.7	11	4.4	0.0936
No	115	91.3	241	95.6	
Exposure to chemicals					
Yes	3	2.4	12	4.8	0.2731
No	123	97.6	240	95.2	
Diabetes					
Yes	15	11.9	15	6.0	0.0476
No	111	88.1	237	94.0	
Radio therapy					
Yes	2	1.6	1	0.4	0.2555
No	124	98.4	251	99.6	
Thyroid disease in family					
Yes	5	4.0	12	4.8	0.7260
No	121	96.0	240	95.2	
Malignancies in family					
Yes	16	12.7	25	9.9	0.4141
No	110	87.3	227	90.1	

*Univariate logistic regression analysis.

**Table 3:** Hormonal and reproductive characteristics of female examinees.

	Cases		Controls		P*
	*N*	%	*N*	%	
Menarche before 15					
Yes	61	79.2	140	90.9	0.0151
No	16	20.8	14	9.1	
Normal menstruations					
Yes	73	94.8	149	96.8	0.4747
No	4	5.2	5	3.2	
Pregnancy					
Yes	73	94.8	152	98.7	0.1041
No	4	5.2	2	1.3	
First pregnancy before 19					
Yes	14	19.2	14	9.2	0.0376
No	59	80.8	138	90.8	
Number of children					
<2 children	37	48.1	84	54.4	0.2751
≥2 children	40	51.9	70	45.6	
Miscarriages					
Yes	4	5.5	4	2.6	0.2905
No	69	94.5	148	97.4	
Abortions					
Yes	58	75.4	109	70.6	0.4158
No	19	24.6	45	29.4	
Oral contraceptives					
Yes	20	26.0	34	22.1	0.5099
No	57	74.0	120	77.9	
Menopause estrogens					
Yes	5	7.0	8	5.6	0.6678
No	66	93.0	136	94.4	

*Univariate logistic regression analysis.

**Table 4:** Results of multivariate conditional logistic regression analysis.

Variable	*P*	OR	95% CI
Education	0.0105	1.42	1.09–1.86
Blood group type B	0.0431	2.41	1.03–5.66
Goitre	0.0000	25.33	5.66–126.65
